# Daratumumab as Single Agent in Relapsed/Refractory Myeloma Patients: A Retrospective Real-Life Survey

**DOI:** 10.3389/fonc.2021.624405

**Published:** 2021-03-05

**Authors:** Uros Markovic, Alessandra Romano, Vittorio Del Fabro, Claudia Bellofiore, Anna Bulla, Marina Silvia Parisi, Salvatore Leotta, Massimo Gentile, Clotilde Cangialosi, Iolanda Vincelli, Giuseppe Mineo, Marco Rossi, Massimo Poidomani, Giuseppina Uccello, Cinzia Maugeri, Donato Mannina, Vanessa Innao, Francesco Di Raimondo, Concetta Conticello

**Affiliations:** ^1^ Postgraduate School of Hematology, University of Catania, Catania, Italy; ^2^ Division of Hematology, University Hospital Policlinico Vittorio Emanuele, Catania, Italy; ^3^ Department of General Surgery and Medical-Surgical Specialties, University of Catania, Catania, Italy; ^4^ Unit of Clinical Hematology, Cosenza Hospital, Cosenza, Italy; ^5^ Unitá Operativa Complessa Ematologia, Azienda Ospedaliera Ospedali Riuniti Villa Sofia Cervello, Palermo, Italy; ^6^ Unità Operativa Complessa di Ematologia, Grande Ospedale Metropolitano Bianchi Melacrino Morelli, Reggio Calabria, Italy; ^7^ Unitá Operativa Semplice Dipartimentale Ematologia, Ospedale San Vincenzo, Taormina, Italy; ^8^ Department of Clinical and Experimental Medicine, Magna Græcia University of Catanzaro, Catanzaro, Italy; ^9^ Servizio di Immunoematologia e Medicina Trasfusionale, Ematologia ASP Ragusa, Ragusa, Italy; ^10^ Unità Operativa Complessa Ematologia, Garibaldi Nesima Hospital, Catania, Italy; ^11^ Division of Hematology, Sant’Elia Hospital, Caltanissetta, Italy; ^12^ Division of Hematology, Papardo Hospital, Messina, Italy; ^13^ Division of Hematology, Department of Human Pathology in Adulthood and Childhood, Policlinico “G. Martino”, University of Messina, Messina, Italy

**Keywords:** multiple myeloma, relapsed/refractory, salvage treatment, immunotherapy, daratumumab

## Abstract

**Background:**

The anti-CD38 monoclonal antibody daratumumab is approved as a single agent for the treatment of patients with relapsed/refractory multiple myeloma (RRMM) who received at least three prior lines of therapy, including proteasome inhibitor and immunomodulatory agent. A retrospective multicentric study was designed to evaluate feasibility, tolerability, and efficacy of daratumumab in monotherapy in RRMM.

**Methods:**

This study included 44 consecutive RRMM patients that underwent daratumumab monotherapy after a median number of four prior therapies (range 2–9). Patients were treated in seven Sicilian centers, as part of Sicilian Myeloma Network and three Calabrian centers outside of controlled clinical trials from August 2016 through July 2020.

**Results:**

The regimen was well tolerated with few grade 3–4 haematological and rare non-haematological adverse events, such as pneumonia. Definitive discontinuation was due to disease progression in 25 (57%) patients. Since three patients did not complete at least one full cycle, a total of 41 patients was evaluated for response. Overall response rate was 37%, and the disease control rate (stable disease or better) was high (73%). The best achieved responses within 6 months were very good partial remission or better (27%), partial remission (10%), minimal response (14%) and stable disease (22%). After a median follow up of 7.8 months, median progression free survival (PFS) was 7.2 months and overall survival (OS) 7.8 months. Univariate analysis showed that patients with PR or better after 6 months of therapy had longer median PFS and OS (respectively 29.5 vs 3.6 months, p=0.0001 and 30.6 vs 3.9 months p=0.0001), confirmed by multivariate analysis. Furthermore, standard cytogenetic risk and biochemical relapse type had prolonged median PFS, but not OS (respectively unreached vs 2.6, p=0.03 and 23.9 vs 6.2, p=0.05) in both univariate and multivariate analysis. Additionally, univariate analysis showed that patients treated with carfilzomib-lenalidomide-dexamethasone prior to daratumumab had significantly shorter PFS compared to pomalidomide-dexamethasone (3.4 months vs 9.3 months, p=0.03), that multivariate analysis failed to confirm.

**Conclusions:**

Our findings indicate that daratumumab as single agent is safe and well-tolerated regimen in real-life, associated to prolonged PFS and OS in responding patients. No new safety signals were identified.

## Introduction

Multiple myeloma (MM) is a chronic plasma cells disease characterized by several relapses that require new treatments. Even in the era of novel agents belonging to different classes of mechanism of actions like pomalidomide, carfilzomib, and ixazomib as single agents or in combination regimens, the treatment response remains highly variable. It has been supposed that the progressively shorter duration and lack of response is probably caused by an increasing use of different drugs and their combinations, with growing drug cross-resistance after each relapse (1). Thus, the disease remains incurable in most cases with a constantly growing number of relapsed and refractory multiple myeloma (RRMM) patients in later lines of therapy. The biggest challenge remains the choice of the most suitable salvage therapy in this setting.

Increase in overall survival (OS) in MM patients along with constant therapy improvement, have brought in evidence a new population of frail patients, that could benefit little from the use of novel agents especially in combination, due to their fitness, medical history, previous drug toxicity, adverse events, relapse type, etc (2).

In this setting, recent randomized trials have shown feasibility, sufficient effectiveness and safety of novel drugs as single agents or in combination with dexamethasone in heavily pre-treated RRMM patients, including pomalidomide (MM-002, MM-003) (3, 4), carfilzomib (CHAMPION-1, ENDEAVOR) (5–7), and daratumumab (GEN-501, SIRIUS) (8, 9). On the other hand, elotuzumab (10) and panobinostat (11) did not demonstrate sufficient efficacy as single agents.

Even with very encouraging results with new drugs, the main difference between randomized studies and real-life experience remains the selection of patients. Subjects followed outside of clinical trials often have several comorbidities like impaired kidney, hepatic or heart function, high performance status (PS) score according to Eastern Cooperative Oncology Group (ECOG) and persistent drug toxicity like peripheral neuropathy, recurrent deep vein thrombosis, reduced bone marrow reservoir, etc. This makes hard to personalize the appropriate therapy in advanced stages, estimating not only the disease aggressiveness, but also the patients’ conditions, without data from every-day experience (12).

Daratumumab is a human IgG1 monoclonal antibody that binds CD38-expressing malignant cells with high affinity. It induces tumor cell death through diverse mechanisms of action, including complement-dependent cytotoxicity, antibody-dependent cell-mediated cytotoxicity, antibody- dependent cellular phagocytosis, apoptosis, and to a lesser extent, inhibition of the enzymatic activity of CD38 (13–15). The drug may also target other CD38-expressing immune cells, thereby exerting an immunomodulatory effect relevant not only at diagnosis, but also during subsequent lines of therapy (16, 17). Depletion of regulatory B cells, certain regulatory T cells and myeloid-derived suppressor cells (MDSCs), along with increase of both CD4+ and CD8+, can lead to improved adaptive immune response (18–20).

Monoclonal antibody treatment with daratumumab was available in Calabria for relapsed/refractory multiple myeloma (RRMM) patients since August 2016, and in Sicily in November 2017. The efficacy and safety was evaluated with our real-life experience in heavily pre- treated patients, most of them being unfit and with important comorbidities.

## Methods

### Patient Selection

In this real-life retrospective survey, 44 RRMM patients were treated with salvage regimen based on daratumumab single-agent between August 2016 and July 2020 in seven Sicilian centers (part of the Sicilian Myeloma Network) and three Calabrian centers. Database lock was 31^st^ July 2020. The study was approved by an independent ethics committee of the coordinating center (*Policlinico Catania 1*, n.34/2019/PO) and was conducted in accordance with International Conference on Harmonization Guidelines on Good Clinical Practice and the principles of the Declaration of Helsinki. All patients have provided written informed consent to data recording and collection before being treated with daratumumab. Primary endpoint was the overall response rate (ORR). Secondary endpoints were rate of best responses, time to progression or relapse, progression-free survival, overall survival and safety.

### Procedures and Drug Administration

All patients received daratumumab monotherapy according to the schedule of SIRIUS trial: daratumumab (DARA) 16 mg/kg i.v. per week for 8 weeks (cycles 1 and 2), then every 2 weeks for 16 weeks (up to cycle 6), and every 4 weeks thereafter. First infusion was prepared and divided in two 500 ml diluitions of DARA preceded by standard premedication. The first infusion was started at 50 ml/h, followed by dose escalation up to 200 ml/h, in the absence of infusion-related reactions (IRRs) as manufacturer suggestions. Subsequent infusions were diluited in 500 ml and started from 50 ml/h in second infusion or 100 ml/h in subsequent infusions with an increase up to 200 mL/h. According to the SIRIUS trial, treatment was continued until progression.

To prevent IRRs, patients received premedication 1 h prior to administration of daratumumab as follows: methylprednisolone (100 mg i.v. for the first and second infusion, and 60 mg thereafter in the absence of infusion related reactions (IRRs) during the first two infusions), paracetamol (650–1,000 mg) and diphenhydramine (25–50 mg) or equivalent antihistamine drug, according to SIRIUS trial and local guidelines. Oral methylprednisolone (20 mg) or equivalent was administered for two days after all daratumumab infusions. In order to prevent IRRs, in 21 patients the first infusion of DARA on cycle 1, day 1 was given as a split dose in two days.

Treatment was discontinued in cases of disease progression, unacceptable adverse events or consent withdrawal.

### Concomitant Medications

Seven patients (16%) received treatment with bisphosphonates every 4 weeks during daratumumab treatment. Antibiotic and antiviral prophylaxis was carried out with trimethoprim and sulfamethoxazole (800 mg + 160 mg twice a day, twice a week) and acyclovir 200, 400, or 800 mg daily, according to the policy of each center. Supportive therapy with erythropoietin (EPO) and granulocyte colony-stimulating factor (G-CSF) was administered according to ASH/ASCO guidelines and policy of each single center (21, 22).

### Safety and Efficacy Assessment

Each patient’s medical history was recorded on day 1 of each cycle. Physical examinations were conducted, and blood samples were collected for hematology, renal and liver function tests on day 1 of each cycle and whenever it was considered necessary. Adverse events (23) were graded using the National Cancer Institute Common Terminology Criteria for Adverse Events (CTCAE) (24).

Efficacy assessment was recorded on day 1 starting from cycle 2 and every cycle thereafter. Response evaluation and progression assessment were reported according to International Myeloma Working Group consensus criteria (25), including complete remission (CR, 100% reduction in M protein according to electrophoresis, with negative immunofixation), very good partial response (VGPR, ≥90% reduction in serum M protein, and less than 100 mg urine M protein per day), partial response (PR, ≥50% reduction in serum M protein, and less than 100 mg urine M protein per day), stable disease (SD), progression disease (PD); not valuable (NV). Minimal response (MR) was defined according to European Society for Blood and Marrow Transplantation criteria (26).

According to IMWG criteria, biochemical relapse was defined as an increase of M protein at least 25% from nadir in serum (absolute increase at least ≥0.5 g/l) and/or urine paraprotein (absolute increase at least ≥200 mg/24 h) in 2 consecutive measurements. A 25% increase in the difference between involved and uninvolved free light chain (FLC) with an abnormal ratio and absolute increase of at least 10 mg/dl was also considered as biochemical relapse. On the other hand, clinical relapse was defined as the presence of at least one of the CRAB criteria, namely hypercalcemia, renal insufficiency, anemia and bone lesions (27).

### Statistical Analysis

Descriptive statistics were generated for data analysis and two-sides p-values of 0.05 or less were considered significant. Qualitative results were summarized in counts and percentages. Overall response rate (ORR) was defined as PR or better (CR + VGPR + PR), while disease control (DCR) rate was defined as a response equal or better than stable disease (≥SD).

Descriptive analysis was performed by frequency distribution for continuous variables. Survival analysis were estimated with the Kaplan−Meier method and compared by the log-rank test. The impact of the following factors was evaluated with univariate analysis: age (≤65 years or >65 years), gender, ECOG performance status (<3 or ≥3), number of previous treatment lines (<5 or ≥5), immunoglobulin type (IgG or other), cytogenetic risk (high versus standard risk), previous autologous stem cell transplantation, creatinine clearance level (<60 ml/min versus ≥60 ml/min), baseline hemoglobin level (<10 g/dL versus ≥10 g/dL), baseline lactic acid dehydrogenase level (normal or increased), last treatment line in terms of doublets versus triplets and pomalidomide-dexamethasone versus carfilzomib-lenalidomide-dexamethasone, relapse type (biochemical versus clinical), best response achieved at 6 months of therapy and grade 3/4 hematological adverse events. Cox proportional hazard model was used to assess association between patients, disease characteristics, namely best response achieved at six months, relapse type, last treatment (KRD vs Poma-Dex), and grade 3/4 hematological adverse events, along with progression free survival (PFS); confidence intervals were at 95%. PFS was calculated from the time of daratumumab start until the date of progression, relapse, relapse- related death or date the patient was last known to be in remission. OS was calculated from the start of daratumumab therapy until the date of death for any cause or the date the patient was last known to be alive. PFS and OS were calculated for patients that completed at least one complete 28-day cycle. All calculation were performed using Stat View (CA, USA) and MedCalc version 12.30.0.0 (Producer: MedCalc Software bvba, Ostend (Belgium), www.medcalc.org).

## Results

### Patients’ Characteristics and Treatment

**Figure 1:**
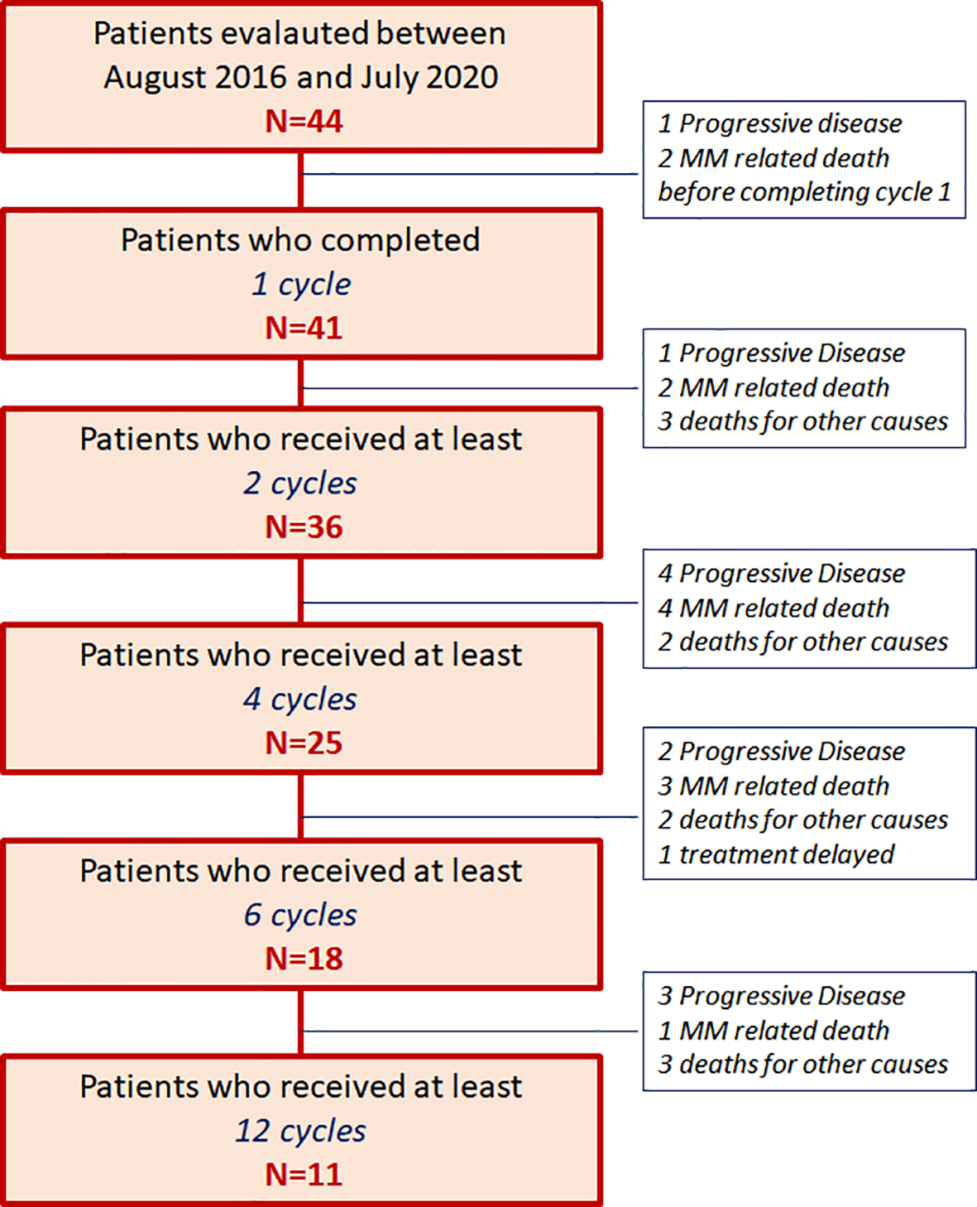
Patients’ allocation [44 patients with relapsed/refractory multiple myeloma (RRMM) patients from August 2016 until July 2020].

This survey included 44 patients treated with daratumumab as single agent outside of clinical trial, from August 2016 until July 2020, and evaluated according to an intention-treat-analysis; 41 patients received at least 1 complete 28-day cycle and were evaluated for efficacy analysis as well (**Figure 1**).

The baseline demographics are summarized in **Table 1**. The median age was 65 years (range 49–82). All patients had measurable disease due to secreted paraprotein; IgG-heavy chain was present in more than half of cases, while in 6 patients the paraprotein was light-chain only.

**Table 1:** Patients’ clinical characteristics in 44 patients with relapsed/refractory multiple myeloma (RRMM) patients treated with daratumumab as single agent [Cytogenetic high risk was defined as the presence of t(4;14), t(14;16) or del17p documented by FISH].

Age	
Median in years (range)	65 (49–82)
< 64 years, N (%)	22 (50)
65–75 years, N (%)	17 (39)
> 75 years, N (%)	5 (11)
**Gender**	
Male, N (%)	24 (55)
Female, N (%)	20 (45)
**Paraprotein (isotype)**	
secreting, N (%)	44 (100)
micromolecolar, N (%)	6 (14)
IgG-heavy chain, N (%)	29 (66)
IgA-heavy chain, N (%)	9 (20)
**Number of prior therapies**	
Median n. of prior therapies, N (range)<5 therapies, N (%)≥5 therapies, N (%)	4 (2–9)29 (66)15 (34)
**ECOG (Performance Status at baseline)**	
0-2, N (%)	30 (68)
3 or more, N (%)	14 (32)
**Risk class at relapse according to IMWG (15 patients)**	
High, N (%)	6 (40)
Standard, N (%)	9 (60)
**Creatinine clearance**	
≥60 ml/min, N (%)	25 (57)
<60 ml/min, N (%)	19 (43)
Dialysis	3 (7)
**Double refractory MM patients (PIs and IMIDs)**	
Yes, N (%)No, N (%)	33 (75%)11 (25%)
**Extramedullary lesions**	
Yes, N (%)	2 (5)
No, N (%)	42 (95)
**Relapse type**	
Biochemical	11 (25)
Clinical (CRAB)	33 (75)

In bold: ECOG Performance Status, Eastern Cooperative Oncology Group Performance Status; IMWG, International Myeloma Working Group; MM, Multiple Myeloma; PIs, Proteasome Inibitors; IMIDs, Immunomodulatory drugs.

At the time of the last relapse, a poor performance status (ECOG score of 3 or more) was present in 14 patients (32%), while three (7%) had impaired renal function (creatinine clearance <30 ml/min), requiring hemodialysis as supportive care. Data on cytogenetic abnormalities, detected by fluorescence in-situ-hybridization (FISH) on highly purified bone marrow plasma cells, were available in 15 patients (34%) at time of relapse, with 6 cases showing a high cytogenetic risk [including del 17p, t(4;14) and t(14;16)].

The median number of prior therapies was four (range 2–9), including 15 (34%) patients who had received five or more. All patients have previously received proteasome inhibitors (PI) and immunomodulatory agents (IMiDs). In most cases, the last regimen received was based on a combination of PI and IMiD (e.g., KRd, 39%) and less frequently on a single novel agent, including pomalidomide (18%), lenalidomide and bortezomib (7% each), or chemotherapy (16%) alone or in associaton with a novel agent. The median time from MM diagnosis to daratumumab monotherapy was 5 years (range 1–22 years). Most patients started immunotherapy suffering from CRAB symptoms at relapse (75%), while only eleven patients were treated for asymptomatic biochemical relapse. Most patients included in the study (75%) were double-refractory to both PIs and IMIDs.

A median number of 6 cycles (range 1–32) per patient was completed; three patients received one incomplete cycle and progressed two died from progression), thus they were excluded from further analysis of efficacy. In one patient daratumumab administration was delayed and reduced due to toxicity and in eight (18%) patients treatment was delayed due to adverse events (in one case not yet recovered), but no definitive discontinuation was recorded.

After a median follow-up of 7.9 months (range 1.1–34.3 months), 12 (27%) patients are still in treatment (11 of them received at least 12 cycle), 10 (23%) patients progressed and shifted to further salvage regimen, 22 (50%) patients died, 12 (27%) for MM progression, and 10 (23%) for other causes: two patients died from myocardial infarction, one from stroke, one sudden intestinal bleeding, one case of pneumonia, two cardiac arrest, three patients from unknown causes, as shown in the patients’ allocation diagram in **Figure 1**.

### Safety

Daratumumab was relatively well tolerated (**Table 1**, **Supplementary Materials**). IRRs were observed in 12 patients (27%), with two of them having more than one episode. In half of these episodes (six patients, 13.5%) grading was 1–2, including short breathness and pruritus, and was safely managed with appropriate supportive care. Severe infusion-related reactions (grade 3 or higher) occurred in remaining patients (six patients, 13.5%) and required temporary suspension (three patients, 7%) or delayed administration (one patient, 2%). All 21 patients who received the first dose split in 2 days, completed the drug infusion as planned without IRRs. Grade 3 or 4 hematological AEs occurred in 14 patients (32%). The most common grade 3–4 hematological AE was anemia, present in 10 patients, and associated with thrombocytopenia in four of them. Nine patients required red blood cell transfusion (20%), and in three (7%) platelet transfusion support was performed. None of the patients developed severe neutropenia, whereas grade 1–2 neutropenia occurred in seven patients (16%). Supportive care with growth factors such as EPO or G-CSF (filgrastim 30 MU) was required in 19 (43%) and seven (16%) patients, respectively, all of them with reduced bone marrow reserve. As for non-hematological AEs, infectious complications were recorded as follows: pneumonia in four patients (9%), severe in two of them (4.5%) requiring hospitalization, fever in four patients, while diarrhea and reactivation of varicella virus (the patient did not assume antiviral prophylaxis) were present in one patient each, respectively. Grade 3 adverse events, both hematological and pneumonia, occurred in the first three months of the treatment, and were recorded only in the patients that did not achieve at least PR (“non responders”). One patient had an atrial fibrillation episode during treatment. No patient undergoing concomitant antibiotic and antiviral prophylaxis had Herpes zoster reactivation or suffered from Pneumocysitis jirovecii related pneumonia.

### Efficacy

Forty-one patients that completed at least one 28-day cycle were evaluated for response (**Table 2**, **Supplementary Materials**). The ORR was 37%, while the disease control rate was high (73%). The best- achieved responses were VGPR+CR in 27%, while 10% attained partial response. In 11 (25%) patients treatment is still ongoing, seven of which achieved at least PR. Median duration of response (DOR) in patients who obtained at least PR (N=15) was 16 (range 4.1–32.3) months, significantly longer than in those not achieving this level of response (N=26), which was 11.5 (range 1–32) months (p=0.04).

**Figure 2:**
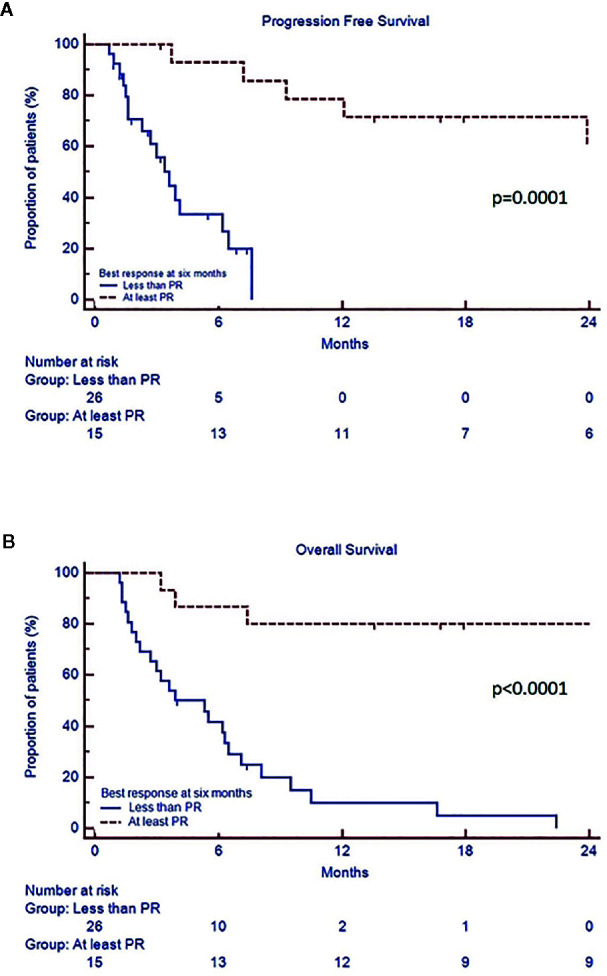
Progression free survival **(A)** and overall survival **(B)** according to best response achieved by 6th cycle.

**Figure 3:**
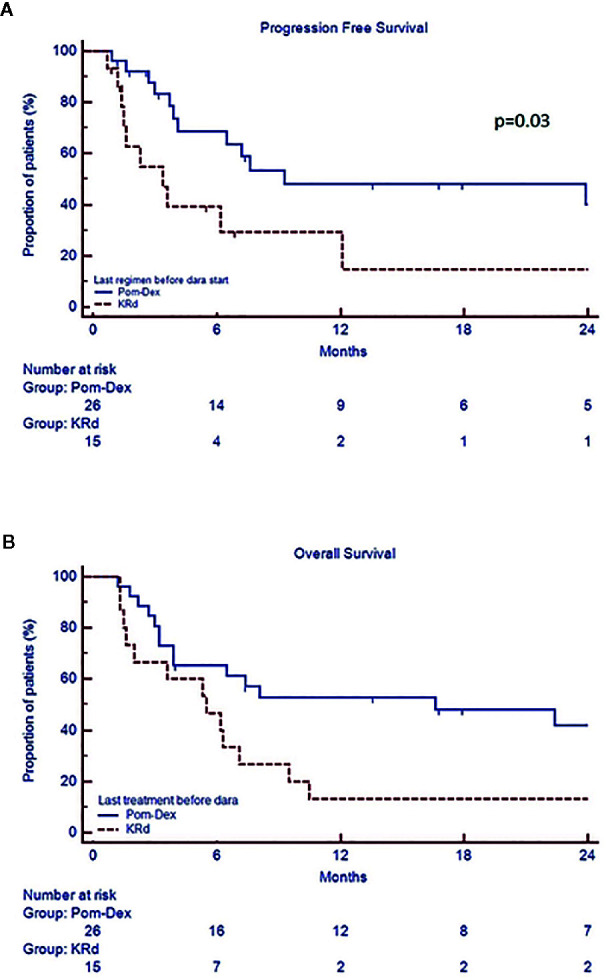
Progression free survival **(A)** and overall survival **(B)** according the type of last therapy (KRd versus Poma-Dex).

In the whole cohort, median PFS was 7.2 months (CI 95% 3.6–29.5) and median OS 7.8 months (CI 95% 3.9–34.3). Univariate analysis showed that patients with PR or better after 6 months of therapy (“responders”) had a prolonged median PFS (range 29.5 vs 3.6 months, p=0.0001) and OS (30.6 vs 3.9 months, p<0.0001) compared to the “non responders”, regardless of the depth of response (**Figure 2**, **Table 3**, **Supplementary Materials**). Both PFS and OS were not affected by age, gender, monoclonal protein type, previous autologous stem cell transplantation, number of prior lines of treatment, baseline LDH, ECOG, creatinine clearance, and last therapy (doublet versus triplet) prior to daratumumab. Standard cytogenetic risk, biochemical relapse type and a previous treatment with pomalidomide-dexamethasone (Poma-Dex), compared to carfilzomib-lenalidomide- dexamethasone (KRd, **Figure 3**), were associated with prolonged median PFS, but not OS (respectively unreached vs 2.6, p=0.03, 23.9 vs 6.2, p=0.05 and 9.3 vs 3.4 months, p=0.03). In addition, despite no differences in PFS, patients who had baseline hemoglobin levels lower than 10 g/dL before daratumumab, had shorter median OS (respectively 5.6 vs 9.5 months, p=0.05) (**Table 3**, **Supplementary Materials**). Most patients with low baseline hemoglobin level did not respond to daratumumab (22 out of 29 patients, 75%). On the other hand, four “responders” with low hemoglobin level eventually recovered along with treatment response in the first 3 months of daratumumab. Patients who experienced hematological adverse events grade 3 or more had inferior PFS, than those who did not (respectively 3.7 vs 9.3 months, p=0.03), without significant difference in OS.

In frail patients, with performance status (PS) ECOG equal or more than 3, both median PFS and OS were shorter compared to patients with PS-ECOG 0-2 (respectively, 4.1 -95% CI 2.7–12.1- versus 7.6 -95% CI 3.7–29.5-, p=0.21 and 4.1 -95% CI 4.7–8.1- versus 9.5 months -95% CI 3.9–30.6-, p=0.09, data not shown).

In multivariate analysis for PFS the following covariates were included: cytogenetic risk, best response achieved at 6 months, relapse type, last treatment (KRD vs Poma-Dex) and grade 3/4 hematological adverse events (**Table 4**, **Supplementary Materials**). High risk cytogenetics and previous treatment with KRd regimen were indipendently associated to shorter PFS (HR respectively 19.2, 95% CI 1.6–233.4, p=0.02 and 15.9, 95% CI 1.6–155.5, p=0.018).

In multivariate analysis for OS, we found that achievement of at least partial remission after 6 months of therapy was associated to longer OS at 24 months.

## Discussion

About five years ago, the introduction of daratumumab as single agent was associated with highly positive results in terms of ORR and median time to response in heavily pre-treated MM patients, with limited toxicities. The accelerated approval of anti-CD38 immunotherapy after phase II clinical trial (9) by both the FDA in November 2015 and EMA in May 2016, opened a new chapter in RRMM management. Soon, the triplet combination with either bortezomib (28) or lenalidomide (29) and dexamethasone demonstrated improved efficacy and response duration, therefore quickly extending the use of daratumumabin combination by FDA in November 2016 and EMA in April 2017. Since then, the broad use of combination therapy has greatly improved both the PFS and OS of RRMM patients, but the use of daratumumab in monotherapy was limited. Therefore, the experience of daratumumab monotherapy in real-life with the exact mechanism of efficacy in population of “responders” (PR or better) is still unknown. In this perspective, retrospective studies outside of clinical trials could help define the population of patients who can benefit from monotherapy with daratumumab, especially in multi-refractory patients with important comorbidities, who are not eligible for combination therapies.

In the drug-approval studies GEN501 and SIRIUS, a total of 148 heavily pretreated patients received daratumumab 16 mg/kg, with a median follow-up of 20.7 months (range, 0.5–27.1 months), as shown in **Table 5** in **Supplementary Materials**. Patients had received a median of 5 prior therapies (range 2–14) and 86.5% of patients were double refractory to both a PI and an IMID. The ORR was 31%, with 14% achieving VGPR or better. PFS was 3.4 months (range, 0.03–26.0 months), rising up to 15 months in responding patients with at least PR (30). Although controlled clinical trials aided greatly in improving the experience of drug mechanism and efficacy, patient selection was limited on the basis of age and comorbidities such as severe renal impairment or performance status, in comparison to real-life population.

On the other hand, real-life studies on daratumumab evaluated the efficacy and tolerability in overall population, thus elaborating the drug’s every-day use. Even though different population studies had significant variation in patient size, double-refractory status and follow-up (31–35), ORR and PFS described in the majority of real-life studies were at least equal, if not superior compared to clinical trials (**Table 5**, **Supplementary Materials**) (30).

In this real-life study we retrospectively evaluated the efficacy and tolerability of daratumumab as single agent in 44 RRMM patients from seven Sicilian centers (belonging to the Sicilian Myeloma Network, SMN) and three Calabrian centers from August 2016 until July 2020, outside of controlled clinical trials. The present study population represents the largest real-life cohort of patients on daratumumab monotherapy. Compared to other studies, the ORR was comparable, if not superior (37%, of which VGPR or better in 27% of patients), together with DCR (73%) and PFS (7.2 months) (**Table 5**, **Supplementary Materials**). Interestingly, patients who achieved at least PR by six months (“responders”), regardless of the depth of response, had a significantly prolonged both PFS and OS.

It is known that CD38 is highly expressed on myeloma cells (36), but it is also present on MDSC (myeloid-derived suppressor cells), Treg (regulatory T cell) and regulatory B cells (18). The presence of myeloma cells in the bone marrow causes important modulation of the environment, leading to immune escape through MDSC and Treg immune suppression (37), with NK and T cell immune dysfunction (38). Daratumumab exhibits lytic activity versus myeloma cells through different immunologic mechanisms: antibody-dependent cellular cytotoxicity (ADCC), complement- dependent cytoxicity (CDC), induction of apoptosis through Fc-mediated cross linking and antibody-dependent cellular phagocytosis (ADCP) (13, 14). However, additional immunomodulatory mechanism has been demonstrated through decrease of CD38 positive immunosuppressive regulatory cells, following an increase in both the effector T cell population and T cell receptor clonality.18 Immunomodulatory functions of daratumumab are complex and probably have a continuous influence on bone marrow microenviroment where myeloma cells find their niche, supporting the role of continuative daratumumab treatment. Also in our experience, there is a significant fraction of RRMM patients who are still benefiting of long-term exposure to DARA.

Given the small number of patients we have not found any disease or patient’s characteristic that brings together all these patients. Our data suggest that DARA could be of benefit in patients refractory to pomalidomide, thus aiding in the optimal sequential strategy for RRMM. It can be hypothesized that the immunomodulatory mechanism of IMIDs could help improve the efficacy of daratumumab priming MM cytotoxicity through loss of Ikaros and Aiolos (39). Also, the contrasting influence of PIs and IMIDs on MDSCs in MM microenviroment could further explain inferior response to DARA monotherapy in KRd refractory patients (17). This subgroup analysis represents a novelty among real-life observations in DARA exposed patients. In general, evaluation of the impact of the previous treatment explored in the real-life setting is emerging as a powerful tool to optimize the sequential treatment in MM (40).

The International Myeloma Working Group (IMWG) has discussed previously about the importance of cytogenetic abonormalities by FISH and high risk cytogenetic abnormalities, namely t(4;14), t(14;16) and del(17p), already included in revised International Staging System (R- ISS) (41, 42). Therefore, FISH analysis prior to change in treatment strategy represents a clinically relevant prognostic factor. In the present real-life study cohort even though only a third of subjects had cytogenetic risk status prior to daratumumab, the advantage in terms of PFS was demonstrated by both univariate and multivariate analysis, confirminig the negative prognostic impact of high-risk cytogenetic abnormalities and the opportunity to perform FISH analysis even in relapsed/refractory MM patients.

The importance of early biochemical relapse detection, compared to clinical relapse with end- organ damage in improving subsequent survival and quality of life, has already been described (43), also by our group (Markovic, EHA 2020, abstract n. EP1001). As for our retrospective study, univariate analysis demonstrated statistically significant advantage in terms of PFS in patients treated with daratumumab early at biochemical relapse compared to the clinical, although the advantage was not confirmed in terms of OS and multivariate analysis.

As for the drug’s safety profile, therapy was well tolerated with less than one third of cohort with grade 3-4 hematological (anemia, platelet reduction) and around one fifth having non- hematological AEs, that were in line with the results of GEN501 and SIRIUS (8, 9). Regarding the infectious AEs, compared to other real-life studies, the incidence of grade 3–4 events was lower (31–35). We can hypothesize that the use of antiviral and antibiotic prophylaxis, together with on demand G-CSF supportive therapy could have been of aid in reducing the incidence of infectious complications, similar to our previous real-life experience with Poma-Dex (44) and KRd regimen (40). The presence of grade 3/4 hematological adverse events was also significant in terms of PFS in univariate analysis. However, due to their presence only in “non-responding” patients (less than PR), the benefit was not confirmed in multivariate analysis. It can be presumed that the lack of response led to increased bone marrow failure, thus contributing to hematological toxicity. On the other hand, all “responding” patients (PR or better) resolved their baseline low hemoglobin level (less than 10 g/dL) in the first three months of daratumumab, as mentioned before, thus confirming the importance of tumor burden. Therapy was delayed due to hematological AEs in only one patient, whereas definitive discontinuation in our series was due to disease progression and MM related death. Despite low numbers that could not allow us to understand how performance status at baseline could affect clinical outcome, treatment was also tolerated well in compromised patients with renal insufficiency and PS-ECOG grade 3 or higher than 3, making daratumumab single agent a suitable treatment also in this subset of patients.

The limitations of the study include retrospective observational study design, together with a limited follow-up time. Furthermore, cytogenetic analysis was available in relatively small proportion of patients.

## Conclusions

Our findings indicate that daratumumab as single agent is a safe and well-tolerated regimen in real-life, associated to prolonged PFS and OS in responding patients. No new safety signal was identified. Our real-life results confirmed the efficacy of single-agent daratumumab in advanced patients with RRMM in comparison with data from clinical trials. Achievement of PR within the first six cycles is associated to longer PFS and OS.

Taken together, our data suggest that RRMM patients with standard risk cytogenetics and previous exposure to pomalidomide could have large benefit from long-term exposure to daratumumab.

## Data Availability Statement

The original contributions presented in the study are included in the article/**Supplementary Material**. Further inquiries can be directed to the corresponding authors.

## Ethics Statement

The studies involving human participants were reviewed and approved by Policlinico Catania 1, n.34/2019/PO. The patients/participants provided their written informed consent to participate in this study.

## Author Contributions

All authors have made substantial contributions to all of the following: Project administration: CC. Methodology: AR. UM: interpreted the data and drafted the final article. CC, AR, UM, VDF, CB, AB, MP, SL, MG, CCa, IV, GM, MR, MP, GU, CM, DM, and VI: selected patients, acquired, analyzed, and interpreted the data. CC, AR, and FR: revised the article for important intellectual content and approved the final version for submission. All authors contributed to the article and approved the submitted version.

## Funding

This work was supported by Università degli Studi di Catania, “Fondi di Ateneo 2020-2022, Università di Catania, linea Open Access”.

## Conflict of Interest

CC, FR, AR, and UM received honoraria from Amgen. CC, FR, and AR received honoraria from Celgene.

The remaining authors declare that the research was conducted in the absence of any commercial or financial relationships that could be construed as a potential conflict of interest.

## References

[1] Kumar SK Lee JH Lahuerta JJ Morgan G Richardson PG Crowley J. Risk of progression and survival in multiple myeloma relapsing after therapy with IMiDs and bortezomib: A multicenter international myeloma working group study. Leukemia (2012) 26(1):149–57. 10.1038/leu.2011.196 PMC4109061 21799510

[2] Offidani M Boccadoro M Di Raimondo F Petrucci MT Tosi P Cavo M. Expert Panel Consensus Statement for Proper Evaluation of First Relapse in Multiple Myeloma. Curr Hematol Malig Rep (2019) 14:187–196. 10.1007/s11899-019-00507-x 31077067

[3] Richardson PG Siegel DS Vij R Hofmeister CC Baz R Jagannath S. Pomalidomide alone or in combination with low-dose dexamethasone in relapsed and refractory multiple myeloma: A randomized phase 2 study. Blood (2014) 123(12):1826–32. 10.1182/blood-2013-11-538835 PMC3962162 24421329

[4] Miguel JS Weisel K Moreau P Lacy M Song K Delforge M. Pomalidomide plus low-dose dexamethasone versus high-dose dexamethasone alone for patients with relapsed and refractory multiple myeloma (MM-003): A randomised, open-label, phase 3 trial. Lancet Oncol (2013) 14(11):1055–66. 10.1016/S1470-2045(13)70380-2 24007748

[5] Siegel DS Martin T Wang M Jakubowiak AJ Lonial S Trudel S. A phase 2 study of single-agent carfilzomib (PX- 171-003-A1) in patients with relapsed and refractory multiple myeloma. Blood (2012) 120(14):2817–25. 10.1182/blood-2012-05-425934 PMC4123387 22833546

[6] Berenson JR Cartmell A Bessudo A Lyons RM Harb W Tzachanis W. CHAMPION-1: A phase 1/2 study of once-weekly carfilzomib and dexamethasone for relapsed or refractory multiple myeloma. Blood (2016) 127(26):3360–8. 10.1182/blood-2015-11-683854 PMC4929927 27207788

[7] Dimopoulos MA Moreau P Palumbo A Joshua D Pour L Hájek R. Carfilzomib and dexamethasone versus bortezomib and dexamethasone for patients with relapsed or refractory multiple myeloma (ENDEAVOR): And randomised, phase 3, open-label, multicentre study. Lancet Oncol (2016) 17(1):27–38. 10.1016/S1470-2045(15)00464-7 26671818

[8] Lokhorst HM Plesner T Laubach JP Nahi H Gimsing P Hansson M. Targeting CD38 with daratumumab monotherapy in multiple myeloma. N Engl J Med (2015) 373(13):1207–19. 10.1056/NEJMoa1506348 26308596

[9] Lonial S Weiss BM Usmani SZ Singhal S Chari A Bahlis NJ. Daratumumab monotherapy in patients with treatment-refractory multiple myeloma (SIRIUS): An open-label, randomised, phase 2 trial. Lancet (2016) 387(10027):1551–60. 10.1016/S0140-6736(15)01120-4 26778538

[10] Zonder JA Mohrbacher AF Singhal S van Rhee F Mohrbacher WI Ding H. A phase 1, multicenter, open-label, dose escalation study of elotuzumab in patients with advanced multiple myeloma. Blood (2012) 120(3):552–9. 10.1182/blood-2011-06-360552 PMC4467882 22184404

[11] Wolf JL Siegel D Goldschmidt H Hazel K Bourquelot PM Bengoudifa BR. Phase II trial of the pan-deacetylase inhibitor panobinostat as a single agent in advanced relapsed/refractory multiple myeloma. Leuk Lymphoma (2012) 53(9):1820–3. 10.3109/10428194.2012.661175 22288662

[12] Richardson PG San Miguel JF Moreau P Hajek R Dimopoulos MA Laubach JP. Interpreting clinical trial data in multiple myeloma: translating findings to the real-world setting. Blood Cancer J (2018) 8(11):109. 10.1038/s41408-018-0141-0 30413684 PMC6226527

[13] de Weers M Tai Y-T van der Veer MS Bakker JM Vink T Jacobs DCH. Daratumumab, a Novel Therapeutic Human CD38 Monoclonal Antibody, Induces Killing of Multiple Myeloma and Other Hematological Tumors. J Immunol (2011) 186(3):1840–8. 10.4049/jimmunol.1003032 21187443

[14] Overdijk MB Verploegen S Bögels M van Egmond M van Bueren JJL Mutis T. Antibody-mediated phagocytosis contributes to the anti-tumor activity of the therapeutic antibody daratumumab in lymphoma and multiple myeloma. MAbs (2015) 7(2):311–21. 10.1080/19420862.2015.1007813 PMC4622648 25760767

[15] Lammerts van Bueren J Jakobs D Kaldenhoven N Roza M Hiddingh S Meesters J. Direct in Vitro Comparison of Daratumumab with Surrogate Analogs of CD38 Antibodies MOR03087, SAR650984 and Ab79. Blood (2014). 10.1182/blood.v124.21.3474.3474

[16] Romano A Conticello C Cavalli M Vetro C La Fauci A Parrinello NL. Immunological dysregulation in multiple myeloma microenvironment. BioMed Res Int (2014) 2014:198539. 10.1155/2014/198539 25013764 PMC4071780

[17] Romano A Parrinello NL La Cava P Tibullo D Giallongo C Camiolo G. PMN-MDSC and arginase are increased in myeloma and may contribute to resistance to therapy. Expert Rev Mol Diagn (2018) 18(7):675–83. 10.1080/14737159.2018.1470929 29707981

[18] Krejcik J Casneuf T Nijhof IS Verbist B Bald J Plesner T. Daratumumab depletes CD38+ immune regulatory cells, promotes T-cell expansion, and skews T-cell repertoire in multiple myeloma. Blood (2016) 128(3):384–94. 10.1182/blood-2015-12-687749 PMC4957162 27222480

[19] Feng X Zhang L Acharya C An G Wen K Qiu L. Targeting CD38 suppresses induction and function of T regulatory cells to mitigate immunosuppression in multiple myeloma. Clin Cancer Res (2017) 23(15):4290–300. 10.1158/1078-0432.CCR-16-3192 PMC5540790 28249894

[20] Oostvogels R Jak M Raymakers R Mous R Minnema MC. Efficacy of retreatment with immunomodulatory drugs and proteasome inhibitors following daratumumab monotherapy in relapsed and refractory multiple myeloma patients. Br J Haematol (2018) 183(1):60–7. 10.1111/bjh.15504 PMC6220946 30080247

[21] Rizzo JD Brouwers M Hurley P Seidenfeld J Arcasoy MO Spivak JL. American Society of Clinical Oncology/American Society of Hematology clinical practice guideline update on the use of epoetin and darbepoetin in adult patients with cancer. J Clin Oncol (2010) 28(33):4996–5010. 10.1200/JCO.2010.29.2201 20975064

[22] Aapro MS Bohlius J Cameron DA Dal Lago L Donnelly JP Kearney N. 2010 update of EORTC guidelines for the use of granulocyte-colony stimulating factor to reduce the incidence of chemotherapy-induced febrile neutropenia in adult patients with lymphoproliferative disorders and solid tumours. Eur J Cancer (2011) 47(1):8–32. 10.1016/j.ejca.2010.10.013 21095116

[23] Thanarajasingam G Minasian LM Baron F Cavalli F De Claro RA Dueck AC. Beyond maximum grade: modernising the assessment and reporting of adverse events in haematological malignancies. Lancet Haematol (2018) 5(11):e563–98. 10.1016/S2352-3026(18)30051-6 PMC6261436 29907552

[24] National Cancer Institute. Common Terminology Criteria for Adverse Events (CTCAE ) version 4.0. NIH Publ (2009) 4(03):71. 10.1080/00140139.2010.489653

[25] Kumar S Paiva B Anderson KC Durie B Landgren O Moreau P. International Myeloma Working Group consensus criteria for response and minimal residual disease assessment in multiple myeloma. Lancet Oncol (2016) 17(8):e328–46. 10.1016/S1470-2045(16)30206-6 27511158

[26] Kyle RA Rajkumar SV. Criteria for diagnosis, staging, risk stratification and response assessment of multiple myeloma. Leukemia (2009) 23(1):3–9. 10.1038/leu.2008.291 18971951 PMC2627786

[27] Cavo M Rajkumar SV Palumbo A Moreau P Orlowski R Bladé J. International myeloma working group consensus approach to the treatment of multiple myeloma patients who are candidates for autologous stem cell transplantation. Blood (2011) 117(23):6063–73. 10.1182/blood-2011-02-297325 PMC3293742 21447828

[28] Spencer A Lentzsch S Weisel K Avet-Loiseau H Mark TM Spicka I. Daratumumab plus bortezomib and dexamethasone versus bortezomib and dexamethasone in relapsed or refractory multiple myeloma: Updated analysis of CASTOR. Haematologica (2018) 103(12):2079–87. 10.3324/haematol.2018.194118 PMC6269293 30237264

[29] Dimopoulos MA San-Miguel J Belch A White D Benboubker L Cook G. Daratumumab plus lenalidomide and dexamethasone versus lenalidomide and dexamethasone in relapsed or refractory multiple myeloma: Updated analysis of POLLUX. Haematologica (2018) 103(12):2088–96. 10.3324/haematol.2018.194282 PMC6269302 30237262

[30] Usmani SZ Weiss BM Plesner T Bahlis NJ Belch A Lonial S. Clinical efficacy of daratumumab monotherapy in patients with heavily pretreated relapsed or refractory multiple myeloma. Blood (2016) 128(1):37–44. 10.1182/blood-2016-03-705210 27216216 PMC4937359

[31] Byun JM Yoon SS Koh Y Kim I Jo J Park H. Daratumumab monotherapy in heavily pretreated Asian patients with relapsed and refractory multiple myeloma: A Real-world Experience. Anticancer Res (2019) 39(9):5165–70. 10.21873/anticanres.13712 31519629

[32] Jullien M Trudel S Tessoulin B Mahé B Dubruille V Blin N. Single-agent daratumumab in very advanced relapsed and refractory multiple myeloma patients: a real-life single-center retrospective study. Ann Hematol (2019) 98(6):1435–40. 10.1007/s00277-019-03655-5 30874850

[33] Minarik J Pour L Maisnar V Spicka I Jungova A Jelinek T. Single agent daratumumab in advanced multiple myeloma possesses significant efficacy even in an unselected “real-world” population. BioMed Pap (2019) 163(3):279–83. 10.5507/bp.2018.064 30397362

[34] Park SS Eom HS Kim JS Koh Y Choi CW Lee JJ. Brief report: Clinical experiences after emergency use of daratumumab monotherapy for relapsed or refractory multiple myeloma in real practice. Jpn J Clin Oncol (2019) 49(1):92–5. 10.1093/jjco/hyy177 30476124

[35] Salomon-Perzyński A Walter-Croneck A Usnarska-Zubkiewicz L Dytfeld D Zielińska P Wojciechowska M. Efficacy of daratumumab monotherapy in real-world heavily pretreated patients with relapsed or refractory multiple myeloma. Adv Med Sci (2019) 64(2):349–55. 10.1016/j.advms.2019.05.001 31125864

[36] Lin P Owens R Tricot G Wilson CS. Flow Cytometric Immunophenotypic Analysis of 306 Cases of Multiple Myeloma. Am J Clin Pathol (2004). 10.1309/74r4-tb90-buwh-27jx 15080299

[37] Feyler S Von Lilienfeld-Toal M Jarmin S Marles L Rawstron A Ashcroft AJ. CD4+CD25+FoxP3+ regulatory T cells are increased whilst CD3+CD4-CD8 -αβTCR+ Double Negative T cells are decreased in the peripheral blood of patients with multiple myeloma which correlates with disease burden. Br J Haematol (2009) 144(5):686–95. 10.1111/j.1365-2141.2008.07530.x 19133978

[38] Dosani T Carlsten M Maric I Landgren O. The cellular immune system in myelomagenesis: NK cells and T cells in the development of myeloma [corrected] and their uses in immunotherapies. Blood Cancer J (2015) 5(4):e306. 10.1038/bcj.2015.32 25885426 PMC4450330

[39] Fedele PL Willis SN Liao Y Low MS Rautela J Segal DH. IMiDs prime myeloma cells for daratumumab- mediated cytotoxicity through loss of ikaros and aiolos. Blood (2018) 132(20):2166–78. 10.1182/blood-2018-05-850727 30228232

[40] Conticello C Romano A Del Fabro V Martino EA Calafiore V Sapienza G. Feasibility, Tolerability and Efficacy of Carfilzomib in Combination with Lenalidomide and Dexamethasone in Relapsed Refractory Myeloma Patients: A Retrospective Real-Life Survey of the Sicilian Myeloma Network. J Clin Med (2019) 8(6):877. 10.3390/jcm8060877 PMC6617295 31248142

[41] Sonneveld P Avet-Loiseau H Lonial S Usmani S Siegel D Anderson KC. Treatment of multiple myeloma with high-risk cytogenetics: A consensus of the International Myeloma Working Group. Blood (2016) 127(24):2955–62. 10.1182/blood-2016-01-631200 PMC4920674 27002115

[42] Palumbo A Avet-Loiseau H Oliva S Lokhorst HM Goldschmidt H Rosinol L. Revised international staging system for multiple myeloma: A report from international myeloma working group. J Clin Oncol (2015) 33(26):2863–9. 10.1200/JCO.2015.61.2267 PMC4846284 26240224

[43] Chakraborty R Liu HD Rybicki L Tomer J Khouri J Dean RM. Progression with clinical features is associated with worse subsequent survival in multiple myeloma. Am J Hematol (2019) 94(4):439–45. 10.1002/ajh.25415 30663805

[44] Parisi MS Leotta S Romano A Del Fabro V Martino EA Calafiore V. Clinical Benefit of Long-Term Disease Control with Pomalidomide and Dexamethasone in Relapsed/Refractory Multiple Myeloma Patients. J Clin Med (2019) 8(10):1695. 10.3390/jcm8101695 PMC6832641 31623097

