# Supplementary material for: Daratumumab as Single Agent in Relapsed/Refractory Myeloma Patients: A Retrospective Real-Life Survey
Source: Front Oncol. 2021 Mar 5;11:624405. doi: 10.3389/fonc.2021.624405 (PMC7982826; doi:10.3389/fonc.2021.624405)
Supplement: Supplementary file 1 [file DataSheet_1.zip › Supplementary Table 1.docx]

**Table S1.** Tolerability, treatment exposure and adverse events in 44 RRMM patients treated with daratumumab as single agent.

| **Exposure/Tolerability** |  |
| --- | --- |
| Median duration, 28-day cycles (range) | 6 (1-32) |
| Dose reduction and delay, N (%) | 1 (2) |
| Temporary interruption or delay, N (%)  Definitive discontinuation, N (%) | 8 (18)  25 (57) |
| Deaths (no treatment or disease-related), N (%)  Disease-related deaths, N (%) | 10(23)  24 (54) |
| **Hematological adverse events (grade 3-4),** N (%) | 14 (32) |
| Anemia, N (%) | 10 (23) |
| Thrombocytopenia, N (%) | 4 (9) |
| **Non-hematological adverse events (grade 3-4),** N (%)  IRRs  Pneumonia | 8 (18)  6 (13.5)  2 (4.5) |

Abbreviations: RRMM - Relapsed/refractory multiple myeloma; IRRs – infusion related reactions.
