# Supplementary material for: Daratumumab as Single Agent in Relapsed/Refractory Myeloma Patients: A Retrospective Real-Life Survey
Source: Front Oncol. 2021 Mar 5;11:624405. doi: 10.3389/fonc.2021.624405 (PMC7982826; doi:10.3389/fonc.2021.624405)
Supplement: Supplementary file 1 [file DataSheet_1.zip › Supplementary Table 2.docx]

**Table S2.** Evaluation of efficacy of daratumumab in 41 RRMM patients (three patients were not evaluated)**.**

|  | **Within first 6 cycles**  **N (%)** | **Best response**  **N (%)** |  |
| --- | --- | --- | --- |
| **CR** | 2 (5) | 2 (5) | ORR 37%  DCR 73% |
| **VGPR** | 9 (22) | 9 (22) |  |
| **PR** | 4 (10) | 4 (10) |  |
| **MR** | 6 (14) | 6 (14) |  |
| **SD** | 9 (22) | 9 (22) |  |
| **PD** | 11 (27) | 11 (27) |  |
| **NE (< 1 cycle)** | 3 | 3 |  |

Abbreviations: RRMM – Relapsed/refractory multiple myeloma; CR – Complete response; VGPR – Very good partial response; PR – Partial response; MR – Minimal response; SD – Stable disease; PD – Progressive disease; NE – not evaluated; ORR – Overall response rate; DCR – Disease control rate.
