# Supplementary material for: Daratumumab as Single Agent in Relapsed/Refractory Myeloma Patients: A Retrospective Real-Life Survey
Source: Front Oncol. 2021 Mar 5;11:624405. doi: 10.3389/fonc.2021.624405 (PMC7982826; doi:10.3389/fonc.2021.624405)
Supplement: Supplementary file 1 [file DataSheet_1.zip › Supplementary Table 3.docx]

**Table S3.** Univariate analysis of PFS and OS in 41 RRMM patients treated with daratumumab as single agent.

|  | *Category* | *n.* | *median PFS*  *(95% CI), months* | *p-value* | *median OS*  *(95% CI), months* | *p-value* |
| --- | --- | --- | --- | --- | --- | --- |
| Age | <=65 | 19 | 7.2 (6.9-17.8) | 0.87 | 7.2 (6.9-17.8) | 0.87 |
|  | >65 | 22 | 7.6 (7.8-19.9) |  | 4.9 (2.4-15.3) |  |
| Sex | male | 25 | 9.3 (9.5-20.1) | 0.25 | 9.6 (9.5-20.1) | 0.11 |
|  | female | 16 | 6.2 (3.2-16.1) |  | 4.6 (2.2-15.3) |  |
| IgG type | no | 15 | 7.2 (4.6-18.9) | 0.73 | 8.2 (8.5-18.8) | 0.97 |
|  | yes | 26 | 9.3 (8.9-18.9) |  | 4.8 (2.4-15.3) |  |
| Cytogenetic risk | standard | 8 | Unreached | ***0.03*** | 11.9 (8.8-23.2) | 0.22 |
|  | high | 5 | 2.6 (1.4-7.2) |  | 6.2 (3.7-18.3) |  |
| Previous ASCT | no | 15 | 6.2 (3.7-18.3) | 0.49 | 6.2 (3.7-18.3) | 0.76 |
|  | yes | 26 | 9.3 (8.3-19.3) |  | 6.9 (4.4-18.3) |  |
| Baseline hemoglobin | <10 g/dL | 23 | 6.5 (5.1-16.6) | 0.31 | 5.6 (3.4-17.1) | ***0.05*** |
|  | ≥10 g/dL | 18 | 12.1 (9.1-20.1) |  | 9.5 (4.2-28.6) |  |
| Baseline LDH | normal | 24 | 9.3 (8.9-20.1) | 0.33 | 6.6 (4.4-23.1) | 0.39 |
|  | increased | 17 | 6.5 (4.7-16.6) |  | 5.4 (3.4-17.1) |  |
| ECOG | <3 | 28 | 7.6 (9.5-19.8) | 0.21 | 8.4 (3.7-24.5) | 0.07 |
|  | ≥3 | 13 | 4.1 (23-14.3) |  | 5.6 (1.6-7.9) |  |
| CrCl | <60 ml/min | 16 | 12.1 (8.9-22.9) | 0.39 | 6.4 (5.4-16.4) | 0.82 |
|  | ≥60ml/min | 25 | 6.2 (5.3-16.2) |  | 6.4 (5.3-16.2) |  |
| Relapse type | Biochemical | 11 | 23.9 (3.1-29.5) | ***0.05*** | 13.1 (2.9-29.5) | 0.09 |
|  | Clinical | 30 | 6.2 (2.7-12.1) |  | 8.1 (9.6-18.6) |  |
| Prior lines | < 5 | 28 | 12.1 (3.6-29.5) | 0.28 | 8.4 (9.1-21.9) | 0.11 |
|  | ≥ 5 | 13 | 4.1 (2.7-9.3) |  | 5.6 (3.4-17.1) |  |
| Last therapy | Doublets | 15 | 23.9 (4.1-25.1) | 0.28 | 10.7 (3.4-27.9) | 0.15 |
|  | Triplets | 20 | 7.2 (3.6-29.5) |  | 6.3 (4.5-16.6) |  |
| Last therapy | Poma-Dex | 26 | 9.3 (4.1-29.5) | ***0.03*** | 16.6 (3.9-34.3) | 0.07 |
|  | KRd | 15 | 3.4 (1.4-12.1) |  | 5.5 (1.5-7.1) |  |
| Best response after 6 cycles | less than PR | 26 | 3.6 (1.6-7.6) | ***0.0001*** | 3.9 (2.2-22.4) | ***<0.0001*** |
|  | PR or better | 15 | 29.5 (9.3-29.5) |  | 30.6 (7.4-34.3) |  |
| Grade 3/4 hematological AEs | yes | 10 | 3.7 (0.7-25.1) | ***0.03*** | 6.5 (3.7-34.3) | 0.95 |
|  | no | 31 | 9.3 (3.6-29.5) |  | 8.1 (1.5-10.1) |  |

Abbreviations: PFS - Progression free survival; OS – Overall survival; RRMM - Relapsed/refractory multiple myeloma; ASCT – Autologous stem cell transplantation; CrCl – creatinine clearance; KRd – Carfilzomib-Lenalidomide-Dexamethasone; PR - Partial response; AEs – Adverse events.
