# Supplementary material for: Daratumumab as Single Agent in Relapsed/Refractory Myeloma Patients: A Retrospective Real-Life Survey
Source: Front Oncol. 2021 Mar 5;11:624405. doi: 10.3389/fonc.2021.624405 (PMC7982826; doi:10.3389/fonc.2021.624405)
Supplement: Supplementary file 1 [file DataSheet_1.zip › Supplementary Table 4.docx]

**Table S4.** Multivariate analysis of PFS and OS in 41 RRMM patients treated with daratumumab as single agent.

|  | **Category** | | **PFS**  **HR**  **(95% CI)** | **p-value** | **OS**  **HR**  **(95%CI)** | **p-value** |
| --- | --- | --- | --- | --- | --- | --- |
| At least PR by 6 months | no | | ***HR: 0.03*** | ***0.04*** | ***0.046*** | ***0.0001*** |
| Cytogenetic risk | High | | ***HR: 19.2***  ***(1.6-233.4)*** | ***0.02*** |  |  |
| Last therapy  KRd vs Poma-Dex | KRd | | ***HR: 15.9***  ***(1.6-155.5)*** | ***0.018*** |  |  |
|  |  |  |  |  |  |  |
| Relapse type | Clinical | | HR: 0.73 | 0.84 |  |  |
| Grade 3/4  hematological AEs | yes | | HR: 0.63 | 0.91 |  |  |

Abbreviations: PFS - Progression free survival; OS – Overall survival; HR – Hazard ratio; PR - Partial response; KRd – Carfilzomib-Lenalidomide-Dexamethasone; Poma-Dex - Pomalidomide-Dexamethasone; AEs – Adverse events.
