# Supplementary material for: Daratumumab as Single Agent in Relapsed/Refractory Myeloma Patients: A Retrospective Real-Life Survey
Source: Front Oncol. 2021 Mar 5;11:624405. doi: 10.3389/fonc.2021.624405 (PMC7982826; doi:10.3389/fonc.2021.624405)
Supplement: Supplementary file 1 [file DataSheet_1.zip › Supplementary Table 5.docx]

**Table S5.** Efficacy of daratumumab in clinical trials and real-world practice

| **Study design** | **Nation** | **N. of patients involved** | **Percentage of double refractory patients** | **ORR**  **(%)** | **Median**  **PFS (months)** | **Median OS (months)** | **References** |
| --- | --- | --- | --- | --- | --- | --- | --- |
| Multicentric,  GEN501 trial |  | 42 | 64 | 30 | 12-mo PFS 22%  *pooled analysis* | 24-mo OS 45%  *pooled analysis* | Lockhorst^8^ |
| Multicentric,  Sirius trial |  | 106 | 95 | 30 |  |  | Lonial^9^ |
|  | South Korea | 21 | 76 | 42 | 6 | NR | Byun^31^ |
|  | France | 41 | 59 | 24 | 1.9 | 6.5 | Jullien^32^ |
|  | Czech Republic | 14 | N.A. | 38.5 | 4.6 | NR | Minarik^33^ |
|  | South Korea | 16 | 44 | 56 | 2.7 | na | Park^34^ |
|  | Poland | 30 | 50 | 43 | 9.5 | 13.8 | Salomon-Perzynski^35^ |
| Multicentric,  Real life survey | Italy | 41  Tot. 44 | 75 | 37 | 7.2 | 7.8 | present study |

Abbreviations: ORR – Overall response rate; PFS - Progression free survival; OS – Overall survival; N.A. – not available.
